# Electroacupuncture ameliorate learning and memory by improving *N*-acetylaspartate and glutamate metabolism in APP/PS1 mice

**DOI:** 10.1186/s40659-018-0166-7

**Published:** 2018-07-06

**Authors:** Ruhui Lin, Long Li, Yingzheng Zhang, Sheng Huang, Shangjie Chen, Jiao Shi, Peiyuan Zhuo, Hao Jin, Zuanfang Li, Weilin Liu, Zhifu Wang, Lidian Chen, Jing Tao

**Affiliations:** 1 Fujian Collaborative Innovation Center for Rehabilitation Technology, Fuzhou, 350122 People’s Republic of China; 2 Baoan People’s Hospital Affiliated to Southern Medical University, Shenzhen, 518000 People’s Republic of China; 3 TCM Rehabilitation Research Center of SATCM, Fuzhou, 350122 People’s Republic of China; 4 National-Local Joint Engineering Research Center of Rehabilitation Medicine Technology, Fuzhou, 350122 People’s Republic of China; 5 College of Rehabilitation Medicine, Fujian University of Traditional Chinese Medicine, Fuzhou, 350122 Fujian People’s Republic of China

**Keywords:** Magnetic resonance spectroscopy (MRS), Electroacupuncture, Learning and memory, *N*-Acetylaspartate (NAA), Glutamate (Glu)

## Abstract

**Objective:**

To explore the precise mechanism of electroacupuncture (EA) to delay cognitive impairment in Alzheimer disease.

**Methods:**

*N*-Acetylaspartate (NAA), glutamate (Glu) and myoinositol (mI) metabolism were measured by magnetic resonance spectroscopy, learning and memory of APP/PS1 mouse was evaluated by the Morris water maze test and the step-down avoidance test, neuron survival number and neuronal structure in the hippocampus were observed by Nissl staining, and BDNF and phosphorylated TrkB detected by Western blot.

**Results:**

EA at DU20 acupuncture significantly improve learning and memory in behavioral tests, up-regulate NAA, Glu and mI metabolism, increase the surviving neurons in hippocampus, and promote the expression of BDNF and TrkB in the APP/PS1 transgenic mice.

**Conclusion:**

These findings suggested that EA is a potential therapeutic for ameliorate cognitive dysfunction, and it might be due to EA could improve NAA and Glu metabolism by upregulation of BDNF in APP/PS1 mice.

## Background

Alzheimer disease (AD), a sever neurodegenerative disease, characterized by a progressive memory and cognitive impairment and affects many people aged over 65 years old. With the world population grows, the incidence of AD and the cost of AD are growing at an ever-increasing rate, and it has become a social issue [1]. It is generally agreed that the neuropathologic hallmarks of AD can be summarized as extracellular deposits of amyloid-β protein (Aβ), intracellular neurofibrillary tangles (NFTs) and neuronal loss in the brain [2, 3]. The early accumulation of Aβ may lead a series of downstream events including neuronal degeneration, then evolved into volume atrophy and cognitive functional damage of the corresponding brain area gradually. The hippocampus plays an important role in cognitive function in the nervous system, and neurons in hippocampus are the main cells responsible for learning and memory [4, 5]. The proliferation and differentiation of neurons have accompany with the process of learning and memory change [6–8]. Studies have shown that decreased neurons in CA1 and CA3 of hippocampus are related to cognitive deficits in those specific region [9–12].

Magnetic resonance spectroscopy (MRS) is an noninvasive method to detect the concentration of metabolites in vivo and it could found the abnormal changes of material metabolism at an early stage of AD [13]. *N*-Acetylaspartate (NAA), an acetylated aspartic acid compound, is synthesized in the mitochondria and located predominantly in neural bodies, dendrites, and axons [14]. As a marker of neuronal, a regional decrease of NAA may be an indication of neuronal malfunctioning due to diminished neuronal density, neuronal cell loss, and partially neuronal dysfunction [15]. Glutamate (Glu) is the most important excitatory neurotransmitter in the central nervous system which could regulate synaptic transmission, neuronal growth and differentiation, synaptic plasticity and learning and memory [16]. Glu is related to the number of available neuronal cells, and variations of Glu highly suggests that physiological or pathological changes in neurons. Myoinositol (mI) is generally considered to be the most valuable indicator to estimate in the early of AD. Brain-derived neurotrophic factor (BDNF) is a member of the neurotrophic factor family and it is the most neurotrophic factor in the body, which is tightly regulated by activity-dependent cellular processes and is strongly linked with mechanisms underlying learning and memory. BDNF activation of tyrosine receptor kinase (TrkB) stimulates intracellular signaling cascades implicated in plasticity. Studies have reported that recognition memory was associated with increased release of BDNF in the hippocampus and cortex [17].

Acupuncture is an impactful treatment to ameliorate cognitive impairment of a wide variety of neurological disorders such as Parkinson’s disease [18] and vascular dementia [19]. Our previous researches have demonstrated that electroacupuncture (EA) at Baihui (DU20) acupoint could significantly improve the cognitive function and revers the aberrant cell death in 4 month-old APP/PS1 double transgenic mice [20], and promote the level of uptake rate of glucose in 12 month-old [21]. But its mechanism remain to be studied. The aim of the present study was to explore the mechanism of EA to improve learning and memory. In this study, learning and memory was evaluated in APP/PS1 transgenic mice by Morris water maze and step-down avoidance test, the material metabolism of the hippocampus were measured by MRS, the expression of BDNF and TrkB were detected by Western blot, and neuronal morphology were observed by Nissl staining.

## Methods

### Animals

A double transgenic *APPSwe/PS1B6C3*-*Tg [B6C3*-*Tg (APPswe, PSEN1dE9)85Dbo/MmJNju]* mouse model was used in this study. Male double transgenic APP/PS1 mice and wild-type mice were obtained from Model Animal Research Center of Nanjing University (SYXK2013-009). 48 mice (12 month-old) were maintained by crossing the male double transgenic mice with female wild-type mice. These mice were gene identification by PCR analysis of genomic DNA from tail biopsies. All mice were male, weighing (25 ± 2) g, housed at 21–25 °C with 60–70% humidity under controlled conditions (12-h light/dark cycle), free to access diet and water. Behavioral tests were conducted by researchers who were blind to grouping. All animal experiments involved ethical and humane treatment under a license from the Fujian Provincial Bureau of Science.

### Experimental protocol

36 mice were randomly divided into three groups, 12 mice in each group: the APP/PS1 double-transgenic mice group (APP/PS1), the EA at Baihui (DU20) acupoint group (APP/PS1+DU20), the EA at non-acupoint group (APP/PS1+NA); and another 12 wild-type littermates were designated as the WT group. After the first time behavioral test, EA stimulation was applied at Baihui point (the intersection of the sagittal midline and the line between the two ears) with 0.5 mm needle inserted at a depth of 5 mm for 4 weeks, once a day, 30 min each time. The stimulation parameters were set as disperse waves of 1 and 20 Hz and the intensity of the stimulation was 1 mA by EA apparatus (model G6805; Suzhou Medical Appliance Factory, Shanghai, China). In the APP/PS1+NA group, the non-acupunct point (the area below the costal region, 2 cm superior to the posterior superior iliac spine and ~ 3 cm lateral to the spine) of the mouse’s left side was selected to accept electric stimulation. The same needles, stimulation parameters and apparatus were used to treat the two groups (APP/PS1+DU20 and APP/PS1+NA). The APP/PS1 group and the WT group fed and grasped in the same conditions without any treatment.

### Behavioral tests

#### Morris water maze test

In order to evaluate learning and memory, Morris water maze test was performed for 5 days as previously described [22]. The water maze apparatus (Chinese Academy of Sciences, Beijing, China) consists of a cylindrical tank (diameter 120 cm, height 50 cm, water depth 30 cm) and a camera (BS-602DVDPLUS 2.0 capture) which connected to a computer. The tank is divided into four quadrants and the platform (diameter 10 cm) is placed in the third quadrant, 2 cm from the surface of the water. During the orientation navigation, a mouse was placed in the water from an equidistant location to the platform in each quadrant for 4 days. When a mouse stayed in the platform more than 3 s within 90 s, the time will be recorded as the escape latency of the mouse in this quadrant. If the mouse did not find the platform in 90 s, it would be removed from water and put in the platform for 15 s to learning. The fifth day is probe trials to investigate the consolidation of memory. The platform was removed and the mouse was put in the first quadrant to swim 90 s. The times of each mouse crossed the original platform area and the swimming trajectory were recorded.

#### Step-down avoidance test

Step-down avoidance test was implement after Morris water maze test to measure the retention of memory, according to described methodology [23, 24]. A mouse was put in a cylindrical insulation platform in a reaction box (20 × 20 × 60 cm) to adapt to the surrounding environment for 3 min. When the mouse stepped down the platform, it would receive an electric stimulated (voltage 32 V, current 0.25 A) for 2 s immediately. In the 5 min training session, the mouse would jump on the platform to avoid stimulation, and some mice might jump repeatedly. 24 h after training, the latency period (stepping down from the platform for the first time) and the number of errors (frequency of jumping off the platform) were recorded.

### Neuroimaging

Magnetic resonance imaging (MRI) and magnetic resonance spectroscopy (MRS) were performed at Fujian University of Traditional Chinese Medicine, using a Bruker Biospec 7.0T (70/20USR MRS scanner, Bruker Biospin, Germany).

#### Magnetic resonance imaging

1.2–1.5% isoflurane and 30% oxygen mixed gas were applied to anesthetize mice, then fixing the mouse head in the center of the special surface coil and monitoring the respiratory rate in real time. The body temperature was maintained at 37 ± 2 °C during the scanning. T1WI (T1-weighted images) scanning was performed from sagittal, axial, and coronal to confirm the position. Then T2WI (T2-weighted images) scanning using the rapid acquisition with relaxation enhancement (RARE) sequence, TR = 2500 ms, TE = 36 ms, FOV = 32 mm × 32 mm, slice thickness = 0.8 mm, IMAGE size = 256 × 256, slices = 15, repeated 8 times, taking 5 min of each mouse.

#### Magnetic resonance spectroscopy

On the basis of the T2WI, volume of interest (VOI) of 1.5 × 1.5 × 1.5 mm^3^ was selected in the left hippocampus. A single volume ^1^H-MRS spectra was acquired with point-resolved echo spin spectroscopy (PRESS) technique with TR = 2500 ms, TE = 16.168 ms, NEX = 1. Water suppression, calibration and shimming were performed by automatic pre-scanning procedure of each voxel.

Quantification of the metabolite concentration was performed using Topspin 6.0 software package. Four dominant signals were analyzed (Fig. 4): *N*-acetylaspartate (NAA) at 2.02 ppm, glutamate (Glu) at 2.2 ppma, myoinositol (mI) at 3.56 ppm and creatine (Cr) at 3.02 ppm. The ratios of NAA/Cr, Glu/Cr and MI/Cr were calculated to express the level of the metabolites in the brain.

### Nissl staining

Mice were sacrificed after MRS scan. 1% Pentobarbital Sodium (701O031, Solarbio, China) was used to anesthetize animals (50 mg/kg) by intraperitoneal injection. The brain was removed and kept in 4 °C environment immediately when the animal was perfused with 0.9% saline (1010110610200, Xilong chemical company, Shanghai, China) solution and 4% paraformaldehyde (20161007, Fuchen chemical reagents factory, Tianjin, China) via the left ventricle (n = 6). After 24 h, the brain samples were taken for paraffin embedding, and cut into 5 μm thick along coronal sections. Slices were put into the oven at 60 °C for 30–40 min, and dehydrated by gradient alcohol, then immersed in Nissl dyeing solution for 20 min. CA1 and CA3 of the hippocampal were observed by microscopy (Nikon TS100, Japan). Image-proplus was used to count the number of neurons in three randomly selected fields.

### Western blotting

The remaining 6 mice in each group were decapitated and the hippocampus was lysated in modified radio immunoprecipitation assay buffer. The mixture was placed in a centrifuge at 4 °C, centrifuged at 14,000 r/min for 5 min, and the bicinchoninic acid (BCA) protein assay kit (23227, Thermo Fisher Scientific, USA) was used to determine the concentration of the extracted protein and calculate the volume of samples after absorbing the supernatant. At first, protein samples (50ug each group) were electrophoresed on SDS–polyacrylamide (dc3512-02, Biomiga, USA) according to the molecular weight of BDNF and TrkB, and then transferred onto PVDF (polyvinylidenefluoride) membranes. Next, incubated BDNF (ab10839, 1: 1000, Abacam, USA), TrkB (ab187041, 1: 500, Abacam, USA), p-TrkB (ab109684, 1: 600, Abacam, USA) and β-actin (20536-1-AP, 1: 8000, Abacam, USA) overnight at 4 °C after blocking 5% skimmed milk at room temperature for 2 h. Subsequently, membranes were incubated horseradish peroxidase labeled secondary antibody (ab97051, 1: 5000, Abcam, USA) at room temperature for 1 h. The protein bands were visualized by enhanced chemiluminescence kit and examined with the Bio-Image Analysis System (Bio-Rad, Hercules, CA, USA). Measuring the optical density, the expression of BDNF protein was presented as the densitometric ratio of proteins to β-actin, and the expression of p-TrkB protein was presented as the densitometric ratio of TrkB.

### Statistical analysis

Date were analyzed using the SPSS20.0 software package, expressed as mean ± SEM. In the Morris water maze test, escape latency statistically difference between groups using repeated measurements of variance analysis, and others were compared by one-way analysis of variance, when P < 0.05 presents the statistically difference was significant.

## Results

### EA could ameliorate the learning and memory impairment in APP/PS1 transgenic mice

**Fig. 1:**
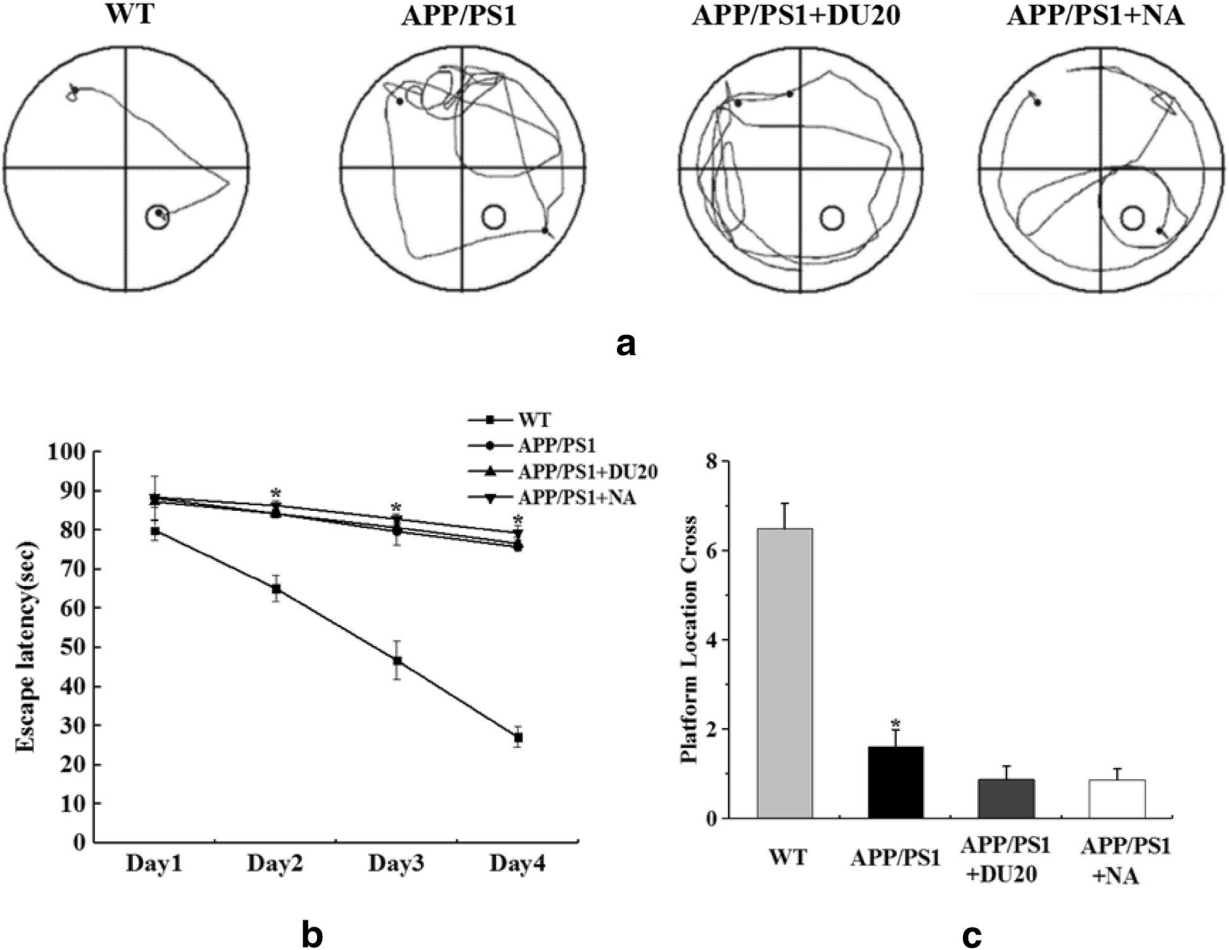
The baseline of different groups in Morris water maze task. **a** Representative swimming paths from different groups. **b** The average latency to discover the hidden platform. **c** Platform location crosses. *P < 0.01 versus WT

**Fig. 2:**
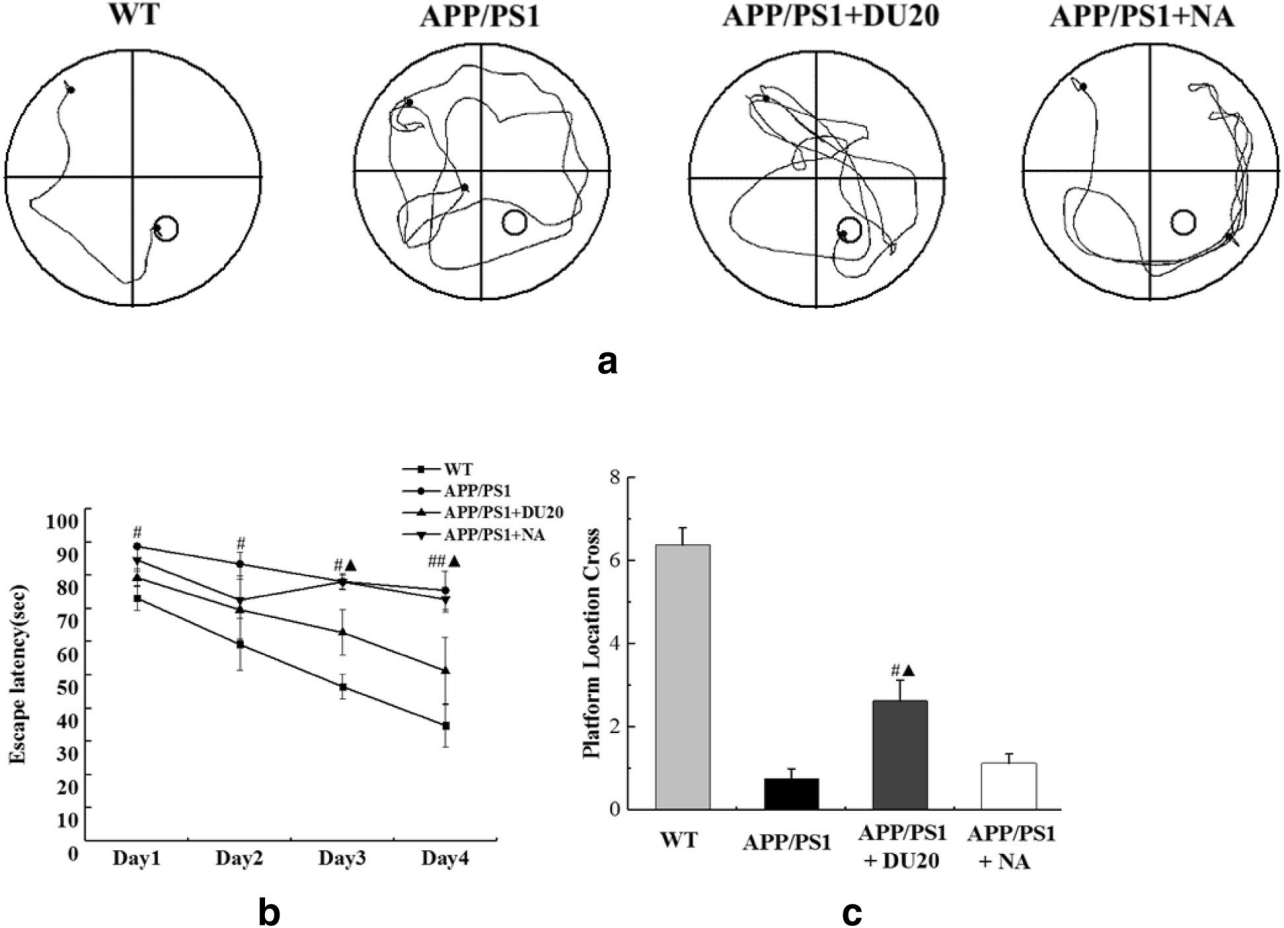
EA at Baihui (DU20) acupoint improve the learning and memory of APP/PS1 mice in Morris water maze task. **a** Representative swimming paths of the different groups. **b** The average latency to discover the hidden platform. **c** Platform location crosses. ^#^P < 0.05 versus APP/PS1 group; ^##^P < 0.01 versus APP/PS1 group; ^▲^P < 0.05 versus APP/PS1+NA group

In order to confirm the efficacy of EA at DU20 acupuncture, the behavioral tests were performed twice. In Morris water maze test, swimming paths of different groups were shown in Figs. 1a, 2a. Compared with the WT group, the APP/PS1 group demonstrated that increased of escape latency (Fig. 1b), and reduced of the number of crossing the platform (Fig. 1c) at the baseline (P < 0.01). No difference was observed in the APP/PS1 group, the APP/PS1+DU20 group and the APP/PS1+NA group (P > 0.05). However, EA at DU20 acupuncture significantly reduced the escape latency compared with the APP/PS1 group (P < 0.05) as shown in Fig. 2b; and increased the number of mice crossing the platform in the probe trials (P < 0.05) as shown in Fig. 2c. Furthermore, there was a difference between the APP/PS1+EA group and the APP/PS1+NA group in the Morris water maze test (P < 0.05). These findings suggested that the learning and memory function was improved by EA at DU20 acupuncture in APP/PS1 double transgenic mice.

**Fig. 3:**
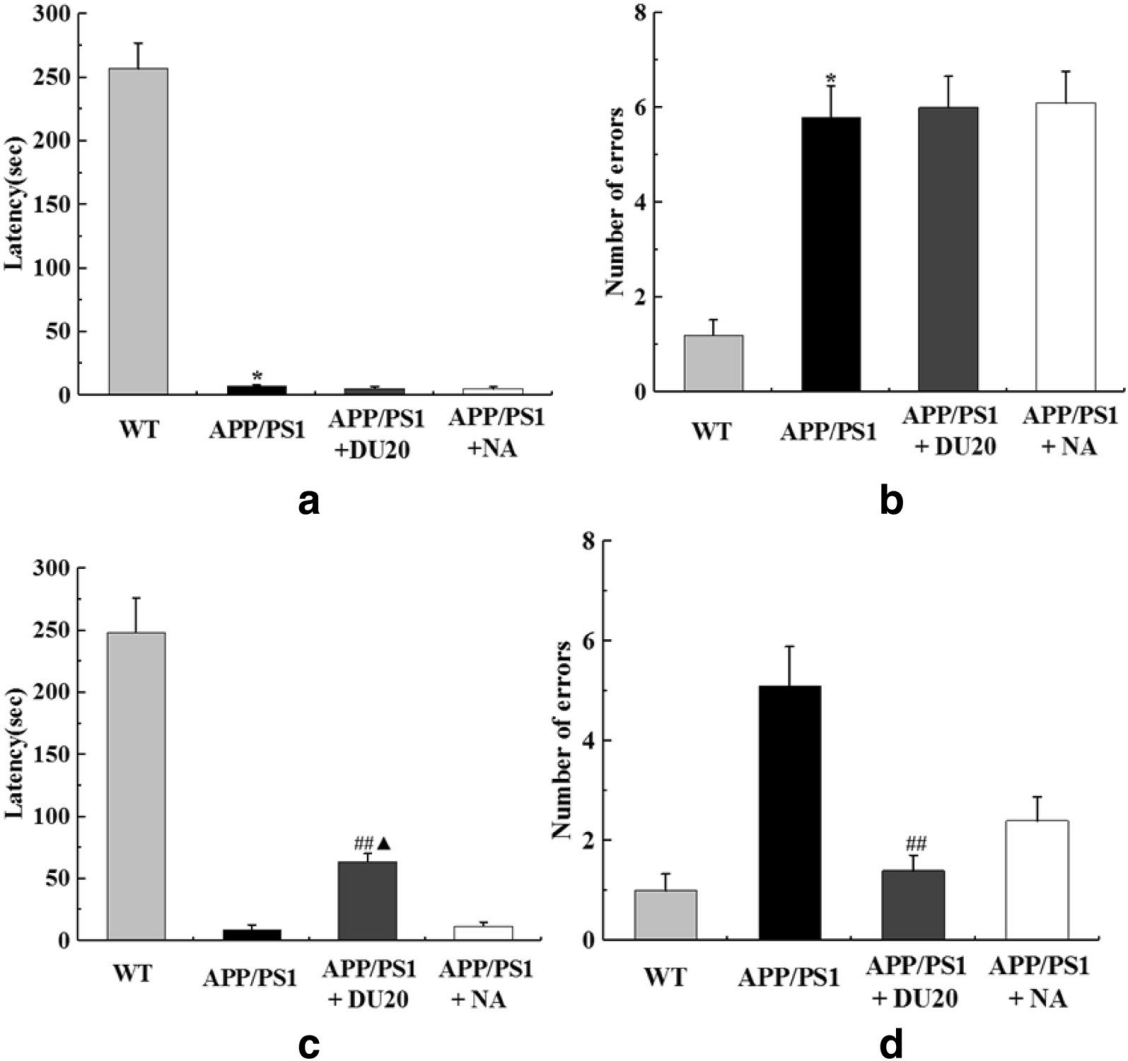
EA at Baihui (DU20) acupoint improved the damaged learning and memory of APP/PS1 double transgenic mice. **a** The latency of different groups at the baseline. **b** The number of errors of different groups at the baseline. **c** The latency of different groups after EA treatment for 4 weeks. **d** The errors of different groups after EA treatment for 4 weeks. *P < 0.01 versus WT; ^##^P < 0.01 versus APP/PS1 group; ^▲^P < 0.05 versus APP/PS1+NA group

In step-down avoidance test, compared with the WT group, both of the latency and the number of errors were markedly higher in the APP/PS1 group (Fig. 3a, b) before EA treatment (P < 0.01). No difference was observed in the APP/PS1 group, the APP/PS1+DU20 group and the APP/PS1+NA group (P > 0.05). Through 4 weeks EA treatment (Fig. 3c, d), latency was longer and the number of errors was less in the APP/PS1+EA groups than these in the APP/PS1 group (P < 0.01); moreover, latency of the APP/PS1+NA group was longer than the APP/PS1+DU20 group (P < 0.05). The results of step-down avoidance test were demonstrated that the damaged learning and memory was improved by EA at DU20 acupuncture in APP/PS1 double transgenic mice.

### The level of NAA and Glu were increased by EA at DU20 acupuncture in APP/PS1 transgenic mice

**Fig. 4:**
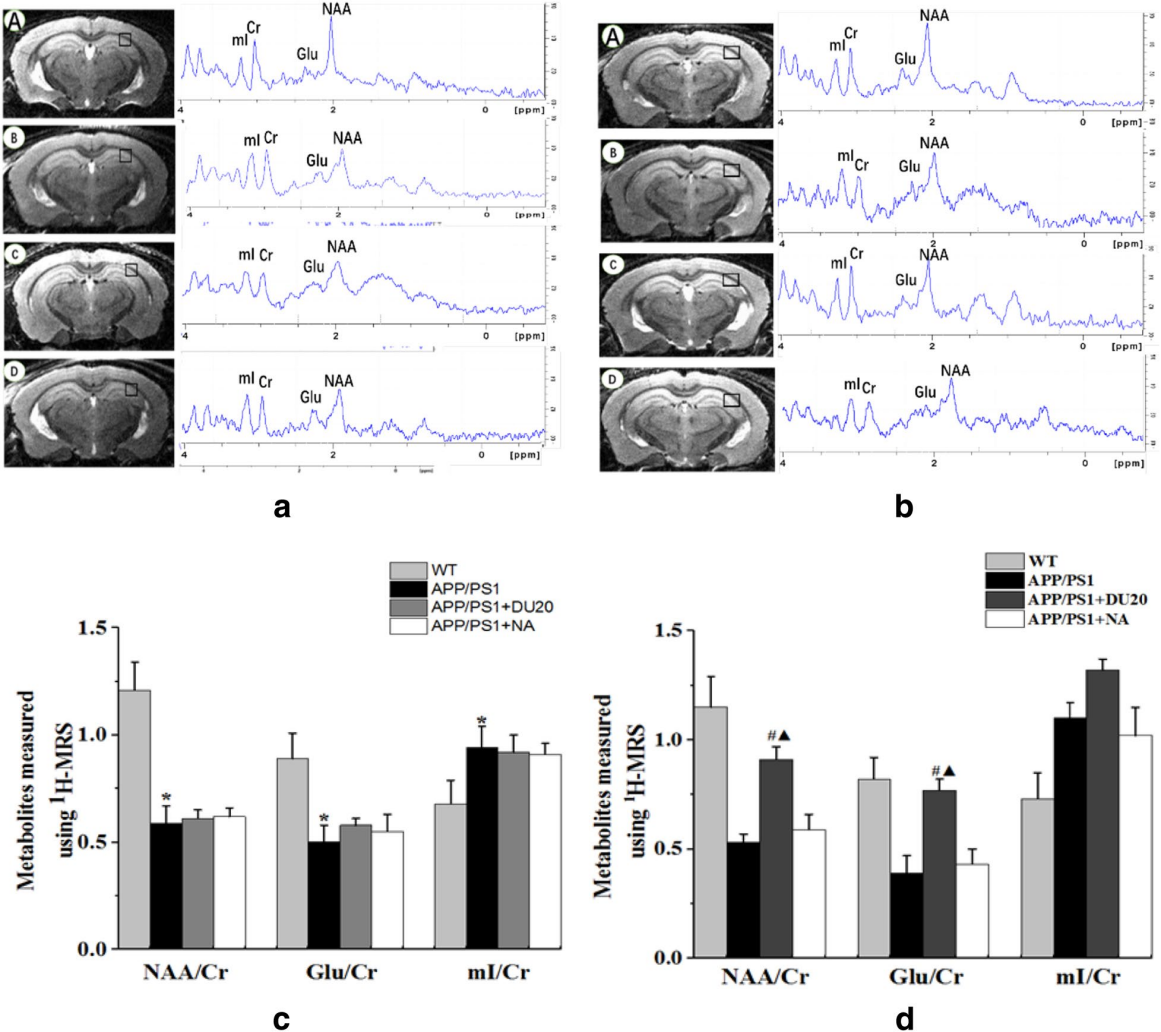
Metabolites measured by using 1H-MRS. **a** The baseline of the 4 groups by using 1H-MRS spectra. **b** NAA/Cr, Glu/Cr, mI/Cr of the 4 groups were detected before EA treatment. **c** NAA/Cr, Glu/Cr, mI/Cr of the 4 groups were detected after EA treatment. **d** NAA/Cr, Glu/Cr, mI/Cr of the 4 groups were detected after EA treatment for 4 weeks. A: WT group, B: APP/PS1 group, C: APP/PS1+DU20 group; D: APP/PS1+NA group. The square represents the detection area. *P < 0.01 versus WT; ^#^P < 0.05 versus APP/PS1 group; ^▲^P < 0.05 versus APP/PS1+NA group

As shown in Fig. 4a, c, the ratios of NAA/Cr, Glu/Cr and mI/Cr in the left hippocampus were selected as VOI by ^1^H-MRS. At the baseline (Fig. 4b), compared with the APP/PS1 group, the contents of NAA/Cr and Glu/Cr were less (P < 0.01), while the content of mI/Cr was higher in the WT group (P < 0.01). These indicated that 12 months old APP/PS1 double transgenic mice had material metabolism changes in the brain. And the level of NAA/Cr, Glu/Cr, mI/Cr were no difference in the APP/PS1 group, the APP/PS1 + DU20 group and the APP/PS1+NA group (P > 0.05). After 4 weeks intervention (Fig. 4d), ^1^H-MRS scan had been shown that NAA/Cr and Glu/Cr in the APP/PS1+EA group were higher than those in the APP/PS1 group (P < 0.05), and there was no difference of mI/Cr in the APP/PS1 group, APP/PS1+EA group and APP/PS1+NA group (P > 0.05). The results of ^1^H-MRS indicated that the material metabolism including NAA, Glu, and mI could be regulated by EA at DU20 acupuncture in the APP/PS1 transgenic mice, and these metabolites which were associated with the pathological of AD.

### The integrity of neurons in CA1 and CA3 were protected by EA at DU20 acupuncture in APP/PS1 transgenic mice

**Fig. 5:**
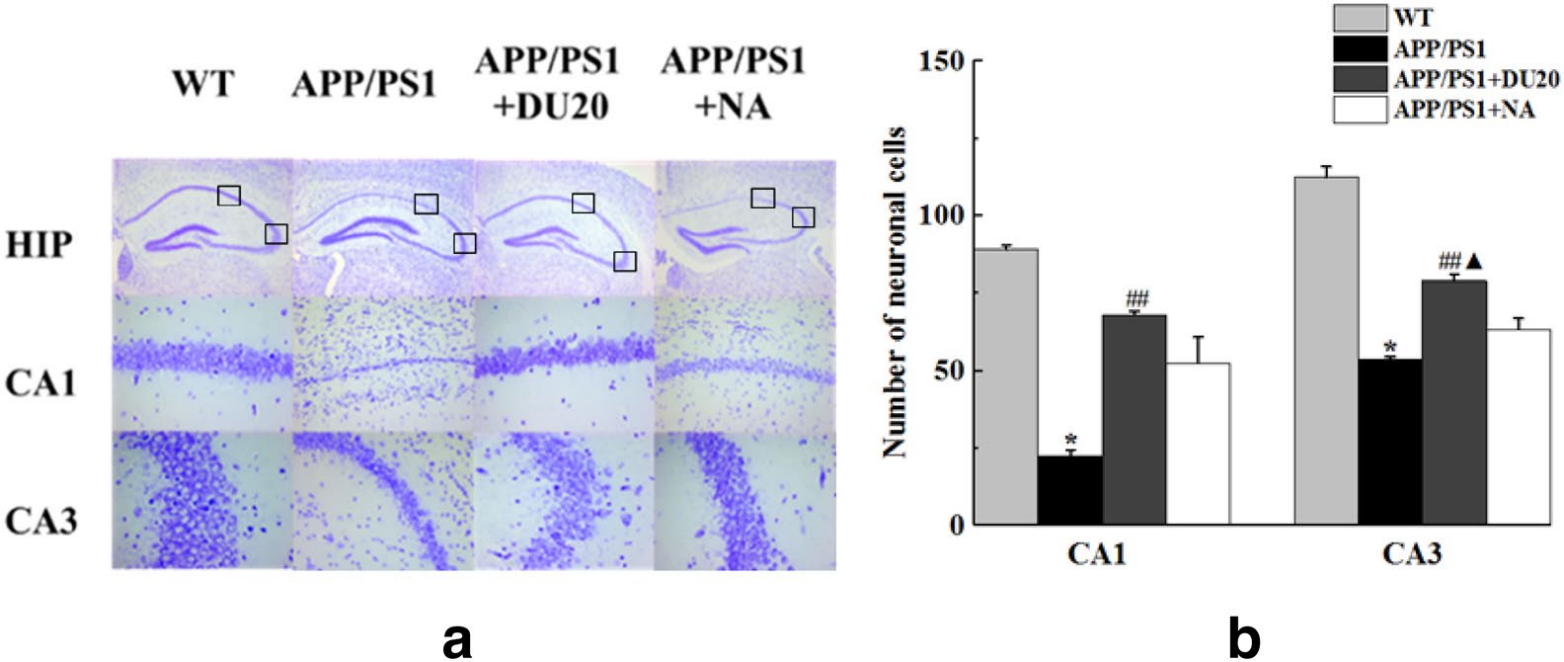
Nissl staining. **a** Hippocampus of Nissl staining of the 4 groups. The first line represent the whole hippocampus, and other pictures represent CA1 and CA3 areas respectively. **b** Number of neuronal cells of the whole hippocampus, CA1 and CA3 areas. *P < 0.01 versus WT; ^##^P < 0.01 versus APP/PS1 group; ^▲^P < 0.05 versus APP/PS1+NA group

Nissl staining was conducted to observe the neuronal morphology and quantity in CA1 and CA3 of hippocampus. As shown in Fig. 5a, the neurons were significantly shrunken and weakly stained in the APP/PS1 group and APP/PS1+NA group, which indicated that neurons were diffusely deteriorated and a great number of Nissl bodies had been lost. While the neuronal cells destruction in the APP/PS1+DU20 group were significantly decrease.

Compared with the WT group (Fig. 5b), the number of surviving neurons of CA1 and CA3 of the APP/PS1 group was significantly decreased (P < 0.01). However, EA could increase the surviving neurons (P < 0.05), and there was no difference of neuronal expression between the APP/PS1+DU20 group and APP/PS1+NA group in CA1(P > 0.05); but the population of surviving neurons was increased in CA3 of the APP/PS1+NA group (*P* < 0.05) compared to the APP/PS1+DU20 group. It is manifested that the number of surviving neurons was increased and their structural integrity in CA1 and CA3 regions were protected by EA in APP/PS1 transgenic mice.

### The expression of BDNF and TrkB were up-regulated by EA in APP/PS1 transgenic mice

**Fig. 6:**
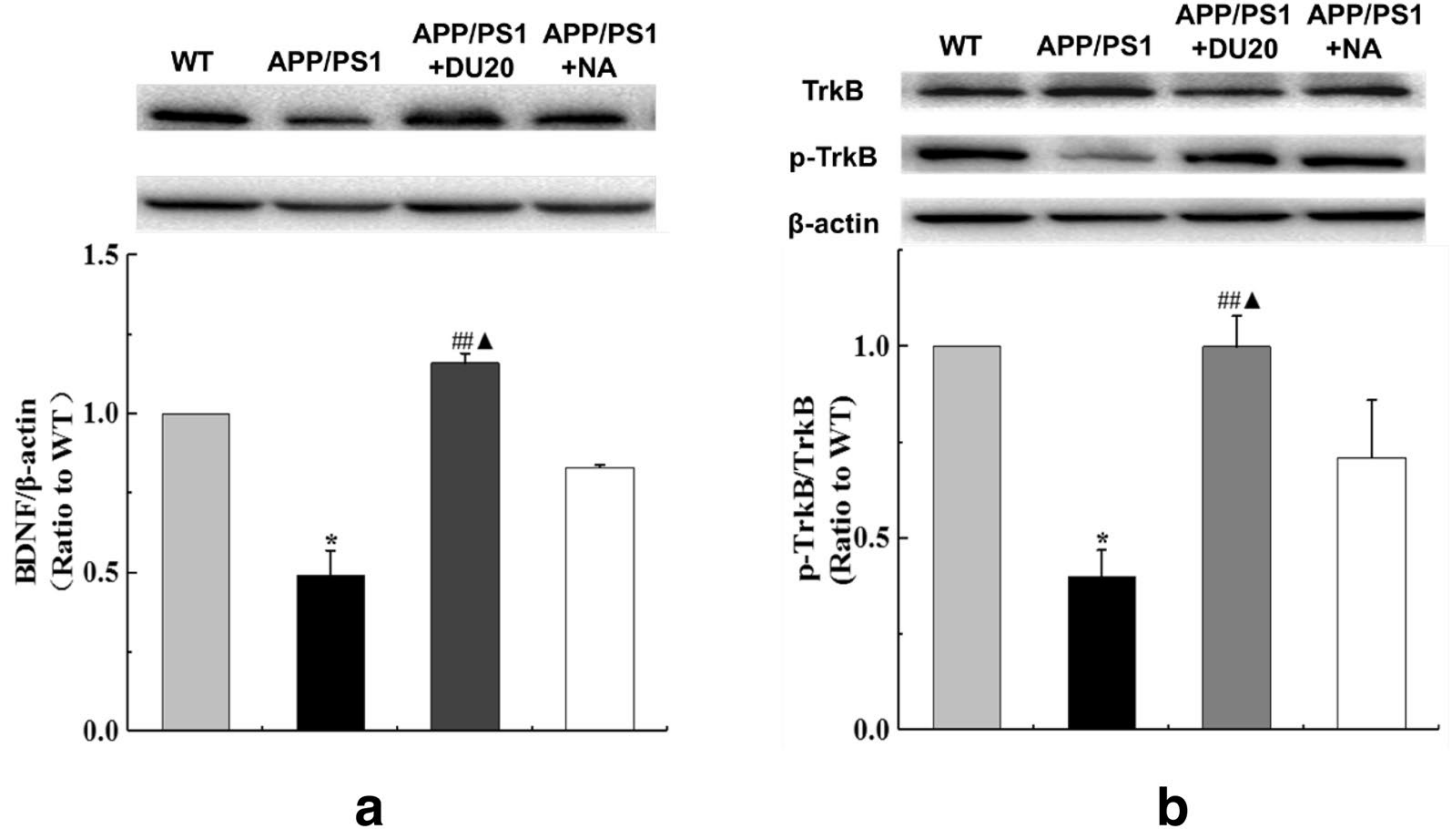
Effects of EA on expression of the BDNF and TrkB of the hippocampus of the APP/PS1 transgenic mice. **a** The expression of the BDNF of the APP/PS1 transgenic mice of the 4 groups. **b** The expression of the TrkB of the APP/PS1 transgenic mice of the 4 groups. BDNF, brain-derived neurotrophic factor; TrkB, tyrosine kinase receptor B; *P < 0.01 versus WT; ^##^P < 0.01 versus APP/PS1 group; ^▲^P < 0.05 versus APP/PS1+NA group

According to the Western Blotting (Fig. 6a, b), the expression of BDNF and TrkB were reduced in APP/PS1 mice compared with the WT mice (P < 0.01); however EA could up-regulate the level of BDNF and TrkB (P < 0.01), and there was a significantly difference between the APP/PS1+DU20 group and the APP/PS1+NA group (P < 0.05). The results of Western Blot shown that EA could promote the expression of BDNF and TrkB, it might be related to promote the number of the neuronal cells or rebuild the function in the penumbra areas.

## Discussion

As the most frequently used, Baihui (DU20) acupuncture, meeting point on the Governing vessel with the six yang channels, some researches suggested that EA at DU20 acupuncture could ameliorate memory-related performance in many behavioral tests as well as modulate cholinergic neurons and improve cerebral blood flow [25–27]. In this present study, Morris water maze test and step-down avoidance test had been shown that the learning and memory were significantly injured in APP/PS1 transgenic mice. We investigated that treatment with EA at DU20 acupuncture could ameliorate behavioral impairment. However, the improvement was not observed in no-acupunct group. The results suggested that EA at DU20 acupuncture had specificity of improve the learning and memory impairment in APP/PS1 transgenic mice.

AD is an age-related disease, and its diagnosis mainly depends on clinical symptoms and neuropsychological assessments, but these can not reflect neuronal changes in the specific region [28]. Therefore, establish a preclinical diagnosis standard before appearance clinically symptom is urgent. MRI plays an important role in the diagnosis of AD, and it could identify subtle pathophysiological changes before brain structure and behavioral abnormal. MRS is a quantitative analysis and imaging technique by specific nuclear chemical shifts [29]. The change of metabolites in the brain may alter the intracellular metabolic environment, tissue structure, and affect cognitive function. We can measure a range of cognitive-related material metabolism in the brain with MRS, which could be used to diagnosis AD and evaluate the physiological and pathological process accurately.

NAA can be used to quantify the damage of neuronal cells [15]; and Glu can reflect the number of surviving neuronal cells. In our study, the contents of NAA and Glu were lower, and mI was higher in the APP/PS1 group than those in the WT group. At the same time, behavioral tests showed that mice had learning and memory disorders with material metabolic changes, and both of those were the result of neuronal absence [21]. However, EA at DU20 acupuncture could increase the level of NAA and Glu, and improve behavioral test of 12 month-old APP/PS1 double transgenic mice. In the Nissl staining, the changes of neuronal cell number and structure in the APP/PS1+DU20 group reflect that EA at DU20 acupuncture has unique advantage to ameliorate the learning and memory impairment in APP/PS1 double transgenic mice. Researches had been demonstrated that NAA and NAA/Cr were significantly decreased in the hippocampus or cortex in APP/PS1 double transgenic mice, and it was thought to be associated with deletion of neuronal cells [30]. Besides, Oberg had been shown that compared with 6–9 months old WT mice, Glu had a significantly reduced in APP/PS1 double transgenic mice [31].

The activation of the BDNF-TrkB signal pathway is necessary for consolidate hippocampus-dependent learning and memory. In our study, the absence and structural damage of neuronal cells resulted in inhibition of BDNF-TrkB in the APP/PS1 group, appearing cognitive impairment which performance as learning and memory dysfunction. The contents of NAA and Glu in the APP/PS1+DU20 group were significantly higher than those in the model group, which promoted the repair of neuronal cell proliferation. Variety of BDNF expression can directly affect NAA concentrations in the brain of patients with degenerative disease [32]. Study had shown that reduced of BDNF mRNA could lead to similarly a low level of NAA, and it is closely related to age-dependent cognitive behavioral decline [33]. In addition, BDNF induced Glu release through activation of the TrkB/phospholipase C-γ (PLC-γ) pathway [34–36]. It indicates that the PLC-γ pathway which activated by the BDNF-TrkB signal is necessary to stimulate Glu release. Use exogenous drugs or other agents to increased TrkB expression, could promote the release of Glu [37]. Moreover, the mechanism of how to EA improve the contents of material metabolism in the brain and increase the expression of related proteins needs to be explored in the future.

## Conclusion

The present study revealed that electroacupuncture at “Baihui” (DU20) acupunct could increase the levels of NAA and Glu, decrease neuronal damage and up-regulate BDNF and p-TrkB in hippocampus. It might be the mechanism underlying EA improve learning and memory and it also provides the foundation for the clinical treatment and research of cognitive dysfunction in the future.
